# Listening to Clinical Teams: Developing Strategies to Support Sustained STAR-VA Implementation

**DOI:** 10.1093/geroni/igab046.2064

**Published:** 2021-12-17

**Authors:** Kim Curyto, Kyle Page, Karen Benson, Laura Wray, Michele Karel

**Affiliations:** 1 VA Western New York Healthcare System, Batavia, New York, United States; 2 Edward Hines Jr VA Hospital, Hines, Illinois, United States; 3 Home Based Primary Care - Kernersville, W.G. Bill Hefner VA Medical Center, Salisbury, North Carolina, United States; 4 VA Center for Integrated Healthcare, Buffalo, New York, United States; 5 Veterans Health Administration, Washington, District of Columbia, United States

## Abstract

Feedback obtained from program evaluations and interviews with CLC team members who participated in STAR-VA helped to inform the development of sustained implementation strategies guided by the CFIR-ERIC Mapping Tool. A CLC readiness assessment was developed to guide selection of new champions and assess for local team readiness to implement STAR-VA. Virtual training materials were developed along with a champion training checklist to prepare additional champions and support team training. We identified key implementation steps and optional strategies to support sustained implementation, developed a sustained implementation guide, associated sustained implementation checklist, and sustainability toolkit. We are piloting a regional community of practice model, encouraging development of and building on relationship networks to promote use of program tools, collaborative problem-solving, feedback, and a shared vision for implementation. We will discuss the importance of tailored strategies for integrating new practices into usual care.

